# Porous tantalum implant for treatment of early-stage osteonecrosis of the femoral head: a minimum 5-year follow-up study

**DOI:** 10.1186/s12893-021-01352-7

**Published:** 2021-10-09

**Authors:** Yu Zhang, Wang Chen, Zhi Yang, Jian-Ning Sun, Zheng-Hao Hu, Zi-Jian Hua, Xiang-Yang Chen, Shuo Feng

**Affiliations:** 1 Department of Orthopedic Surgery, Affiliated Hospital of Xuzhou Medical University, 99 Huaihai Road, Xuzhou, 221002 Jiangsu China; 2 Department of Orthopedic Surgery, The Third Hospital of Hebei Medical University, 139 Ziqiang Road, Shijiazhuang, 050000 Hebei China

**Keywords:** Osteonecrosis of the femoral head, Porous tantalum rod implantation, Survival analysis, Total hip arthroplasty

## Abstract

**Background:**

To evaluate the survival rate of porous tantalum rod implantation in the treatment of osteonecrosis of the femoral head (ONFH), evaluate its clinical effect and imaging results.

**Methods:**

From January 2008 to December 2013, porous tantalum rod implantation for ONFH was performed in two institutions. Statistical analysis of operation data, including operation time, blood loss and blood transfusion were recorded.

**Results:**

52 hips received complete follow-up, the average follow-up time was 85.7 months (60–132 months). 24 hips turned to THA at the end of follow-up (46.2%), the average time was 44.3 ± 32.8 months, and the average Harris hip score before THA was 57.1 ± 7.6. Cox proportional-hazards model revealed that Association Research Circulation Osseous (ARCO) stage (*P* = 0.017), bone marrow edema (*P* = 0.006) and age > 40 years (*P* = 0.043) were independent risk factors for conversion to THA.

**Conclusion:**

ARCO stage, age and bone marrow edema were risk factors for the failure of porous tantalum rod implantation to convert to THA.

## Background

Osteonecrosis of the femoral head (ONFH) is a kind of pathological state with multiple causes, which leads to the decrease of blood supply of the subchondral bone of the femoral head, death of bone cells and collapse of the articular surface [1, 2]. For the early and middle stage of ONFH, the first choice is to retain the femoral head. For the treatment of early ONFH, the main principle is to effectively improve the blood supply of the femoral head and function of the hip, and delay the development of the pathological process of ONFH. At present, several important factors for the treatment of ONFH are as follows: increase new bone regeneration and neovascularization; remove the dead bone thoroughly; reduce the internal pressure of femoral head and promote the internal venous return of hip joint; maintain and increase the surface supporting force of the femoral head, prevent or treat the collapse of the femoral head [3, 4].

Tantalum is a blunt metal with good biocompatibility. Bone and vascular tissue can be seen to grow rapidly in the hip joint with tantalum coating [5, 6]. Core decompression combined with tantalum rod placement can release the internal pressure of the femoral head through core decompression, so as to alleviate the pain. Tantalum metal has the same elastic modulus as bone, and has the structure of bone trabecula, with high porosity. The tantalum rod (Zimmer, United States) has a cylindrical structure with an aperture of 430 μm and a diameter of 10 mm, with a porosity of 75%-80%, and a length of 70–130 mm (5 mm increase). The elastic modulus is equivalent to that of human fibula (3GPa), which has a good support for the femoral head [7]. Tantalum rod can bear physiological load of human body, has good biocompatibility and friction stability. The placement of tantalum rod can play a role in filling the core bone and supporting the femoral head, reducing the stress distribution of the surrounding bone tissue, and effectively preventing the collapse of the femoral head surface. At the same time, the porous structure of tantalum rod can induce osteoblasts to grow in, accelerate the regeneration of blood vessels and promote the process of vascularization, which is conducive to the regeneration and the repair of femoral head.

Many studies have reported that porous tantalum rod implantation has achieved good early clinical effect in the treatment of ONFH [4, 8, 9]. However, there are still controversies regarding the long-stage clinical effect, weight-bearing time and effect of porous tantalum implant [10–12]. In the past few years, the failure rate of tantalum rods in ONFH has been reported ranging from 2 to 56% [8, 13]. Once the subchondral bone collapses, the progression of the disease is difficult to reverse. The collapse and deformation of the femoral head, the narrowing of the joint space, and the deterioration of the joint function occur in turn. Hip replacement has become the only treatment option for these patients.

This study retrospectively analyzed the medium and long-term survival data of ONFH patients treated with porous tantalum rod implantation, and evaluated the clinical and imaging results. The effect of porous tantalum rod implantation on ONFH and the related factors leading to its conversion to THA were analyzed.

## Materials and methods

### Patient selection

From January 2008 to December 2013, patients with ONFH underwent porous tantalum rod implantation in two hospitals were analyzed retrospectively. Inclusion criteria: non traumatic ONFH, Association Research Circulation Osseous (ARCO) I-II patients. Exclusion criteria: skin lesions in the surgical area, active infections, coagulation disorders, and cases previously treated with any other type of treatment. This retrospective study was approved by the ethics committee of our institute, and we confirm that all methods are carried out in accordance with relevant guidelines and regulations. All patients signed the informed consent of operation.

One patient (2 hips) died of diseases unrelated to the operation, and three patients (3 hips) lost follow. A total of 42 patients (52 hips) were finally analyzed in this study. The demographic data and preoperative baseline characteristics of patients are shown in Table 1.

**Table 1:** Demographic and baseline characteristics

Classification	Tantalum rod group
Patients, n	42
Hips, n	52
Age (years)	40.1 ± 9.3 (22–59)
Gender, n(%)	
Female	12 (28.6%)
Male	30 (71.4%)
BMI(Kg/m^2^)	25.6 ± 2.5
Age, n (%)	
> 40 years	33 (63.5%)
≤ 40 years	19 (36.5%)
Bilateral disease, n (%)	30 (57.69%)
Etiology, n (%)	
Corticosteroids	21 (40.38%)
Alcoholism	23 (44.23%)
Idiopathic	8 (15.38%)
ARCO stage, n (%)	
I	22 (42.31%)
II	30 (57.69%)
Bone marrow edema	30 (57.69%)
Osteonecrotic lesion size ≥ 30%, n (%)	27 (51.92%)
Harris hip score	73.4 ± 6.3
Harris hip score, n (%)	
≥ 80 points	10 (19.23%)
< 80 points	42 (80.77%)
Duration of symptoms (months)	9.2 ± 3.7
Follow-up (months)	85.7 ± 16.6 (60–129)

### Surgical methods

The operation were mainly performed by two experienced chief physicians and deputy chief physicians. All surgeons using this device had prior surgical experience and completed their learning curve during the study period. Their surgical techniques have been assessed and approved by institutions.

After anesthesia, the patient was in supine position. The affected hip was placed neutrally in an adducted position. Under fluoroscopic guidance, a guide pin was drilled from the proximal lateral femur into the anterolateral necrotic area of the femoral head. A core reamer was placed over the guide pin to create a 10-mm-diameter bone channel through which necrotic bone tissue and the surrounding hardened area was scraped off by a long curette. A measured tantalum implant (Zimmer, United States) was inserted under fluoroscopic guidance until it abutted the subchondral plate. Fluoroscopy showed that the tantalum bar was in good position, and the incision was sutured layer by layer. The second-generation cephalosporin antibiotics were given to prevent incision infection 30 min before operation and 24 h after operation. In 12 weeks after operation, the patients' hip joint was fully limited in weight-bearing, and functional exercise was performed without weight-bearing. After 12 weeks, they walked with full weight-bearing.

The indications of THA were: continuous hip pain interfered with daily activities and deterioration of hip score, or radiational collapse of femoral head and intraarticular penetration of tantalum rod. The technology of tantalum rod transfer to THA after failure includes femoral neck osteotomy and implant cutting, both of which use a power saw to remove the tantalum rod from the rotor with a special ring drill. This procedure is performed as a routine hip replacement. We used the conventional proximal fixed prosthesis with normal offset, without high offset in this study. And cementless total hip arthroplasty with coating were performed for all surgeries. The prosthesis is tightly integrated with the host bone during the operation, has good anti-rotation type and good stability. Therefore, the weight-bearing situation of these patients undergoing porous tantalum implant in the past and later undergoing THA is the same as that of ordinary patients who underwent THA. If the patient suffers a periprosthetic femoral fracture during the operation, we suggest to postpone the weight-bearing gradually after 6–8 weeks.

### Clinical assessment

All patients underwent clinical and radiographic examinations at one and three months, postoperatively, and at 6-month intervals thereafter. The evaluation parameters included Harris hip score and X-ray and MR examination of the affected hip. The X-ray films of hip joint were used to evaluate the size of lesions, the consistency of femoral head, whether there was crescent sign and the degeneration of hip joint. MR images were used to assess changes in bone marrow edema and lesion size. According to the ARCO [14] grading system, the initial stage and the degree of involvement of the femoral head were evaluated by radiology. Bone marrow edema was defined as a low signal area on T1 weighted images, The high signal region is defined on T2-weighted image or inversion recovery image, in the femoral head, neck and intertrochanteric region [15, 16]. The clinical evaluation was conducted by a non-surgeon observer throughout the study. Two radiologists who were not involved with the operation and blinded to all clinical information, performed radiological measurement. Each parameter is measured twice at appropriate intervals to prevent bias from affecting the results.

### Statistical analysis

The data and charts were analyzed and processed by IBM SPSS Statistical 19.0 statistical software. Continuous variables were analyzed using independent sample T test. Categorical variables were analyzed using the Pearson chi-square or Fisher exact tests. Kaplan–Meier survivorship analyses were used with the endpoint defined as reoperation with THA. We used the Cox proportional hazards model to analyze the independent factors associated with conversion to THA. Test level was set at both sides α = 0. 05, *P* < 0.05 was considered statistically significant.

## Result

### Basic conditions of surgery

The operation was successfully completed in all cases. The mean time was 51 ± 14 min (38–69 min), intraoperative blood loss was 80 ± 21 ml (45–170 ml), and hospital stay was 8 ± 3 days (5–14 days).

### Clinical results

**Fig. 1:**
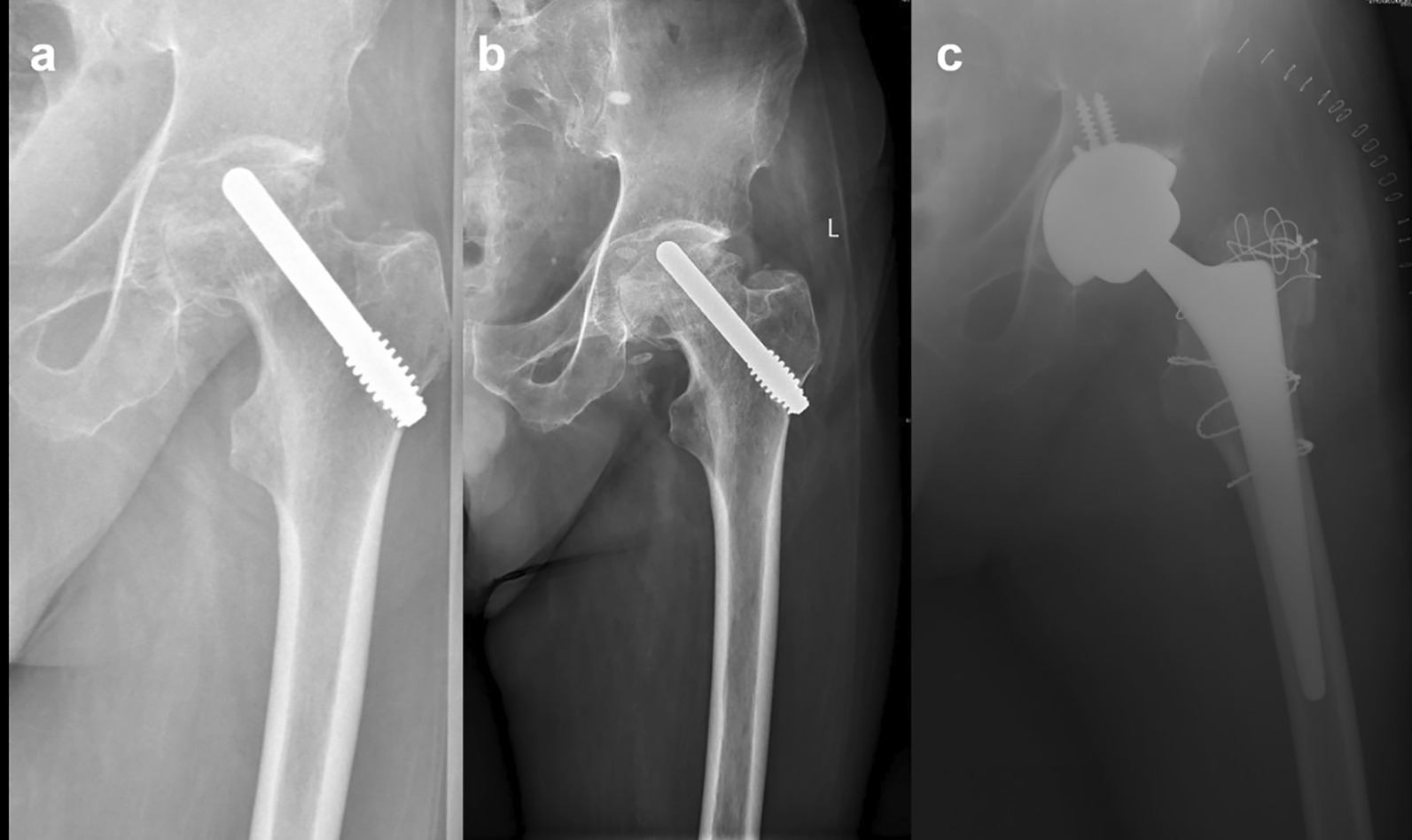
129 months follow-up of a patient with osteonecrosis of the femoral head (ONFH) after porous tantalum rod implantation with conversion to total hip arthroplasty

**Fig. 2:**
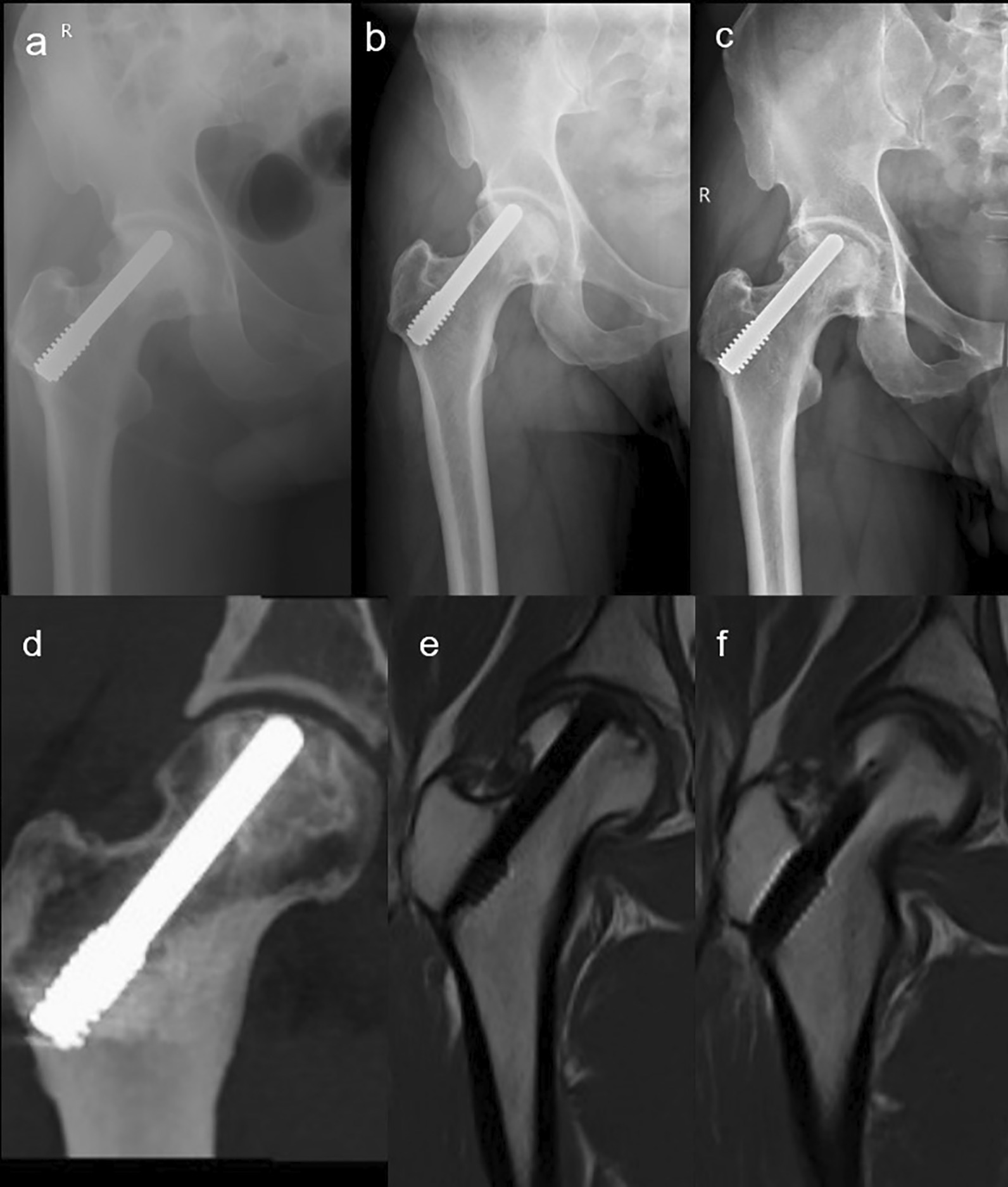
132 months follow-up of a patient with osteonecrosis of the femoral head (ONFH) **a** Postoperative X-ray; **b** 6 years postoperative X-ray; **c** 11 years postoperative X-ray; **d** 6 years postoperative CT; **e**, **f** 11 years postoperative MR

The average follow-up time was 85.7 (60–132) months. The preoperative Harris hip score for all hips were (73.4 ± 6.3). At the last follow-up or before THA conversion, the average Harris hip score for all hips were 69.7 ± 13.2. A total of 24 hips turned to THA at the end of follow-up (46.2%), the average time was 44.3 ± 32.8 months, and the average Harris hip score before THA was 57.1 ± 7.6. (Fig. 1) The follow-up time of 28 hips without THA was 79.4 ± 14.6 months, and the average Harris hip score was 80.6 ± 2.8. (Fig. 2) According to the stage of ARCO before operation, the average Harris hip score at the last follow-up of patients with ARCO I was higher than that of patients with ARCO II (*P* < 0.05); the average Harris hip score of patients with preoperative age ≤ 40 years was significantly higher than that of patients with age > 40 years (*P* > 0.05); there were no significant differences between cases with respect to postoperative Harris hip score in different ONFH etiologies (*P* > 0.05). (Table 2).

**Table 2:** Comparison of postoperative Harris hip scores between different group

Classification		Hips(n)	Last Harris scores	P value
ARCO stage	I	22 (42.31%)	74.0 ± 13.5	0.039*
	II	30 (57.69%)	66.7 ± 12.1	
Bone marrow edema	Yes	30 (57.69%)	64.2 ± 11.3	0.001*
	No	22 (42.31%)	75.3 ± 12.6	
Age	> 40	33 (63.5%)	61.3 ± 10.5	0.000*
	≤ 40	19 (36.5%)	76 .3 ± 11.2	
Etiology	Corticosteroids	21 (40.38%)	68.9 ± 11.9	0.837
	Alcoholism	23 (44.23%)	70.0 ± 14.6	
	Idiopathic	8 (15.38%)	70.1 ± 13.4	

*Denotes significant difference between groups (P < 0 .05)

### Radiographic results

At the last follow-up, 28 of the 52 hips showed deterioration on imaging (53.8%). Among them, 5 of 22 hips in ARCO I stage progressed to stage III and 3 to stage II; 12 of 30 hips in ARCO II stage progressed to stage III and 8 to stage IV. The imaging progress rate of hip joint in ARCO I stage (36.4%; 8/22) was significantly lower than that in ARCO II stage (66.7%; 20/30) (x^2^ = 4.690, *P* < 0.05). Among the 28 hip joints with imaging progress, 24 (89.1%) needed THA finally, while none of the 24 hip joints without imaging progress needed THA.

### Survivorship and factor analysis of transition to THA

**Fig. 3:**
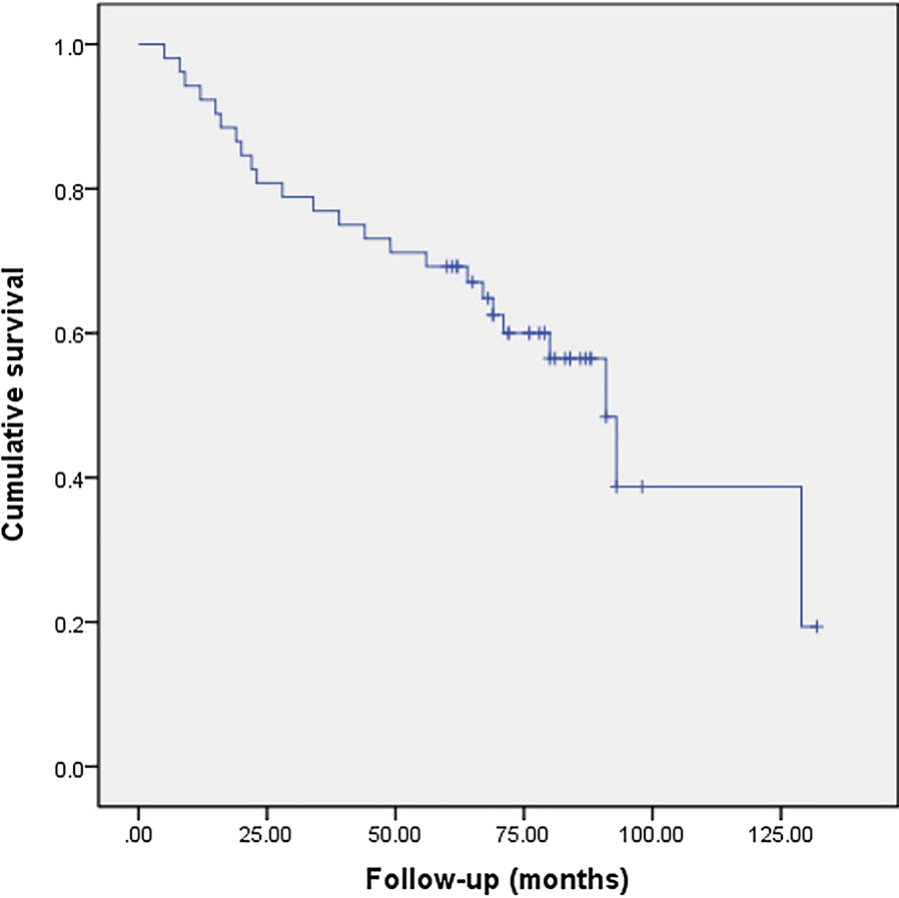
Kaplan–Meier survivorship curve with conversion to total hip replacement

**Fig. 4:**
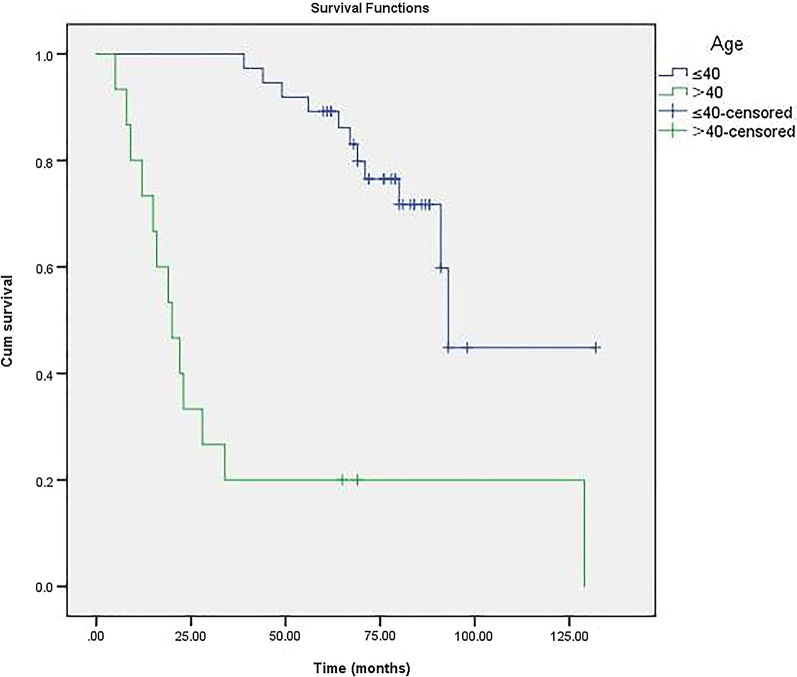
Comparison of survival time between age groups (≤ 40 years and > 40 years). The survival time was significantly shorter in the latter group (*P* < 0.001)

**Fig. 5:**
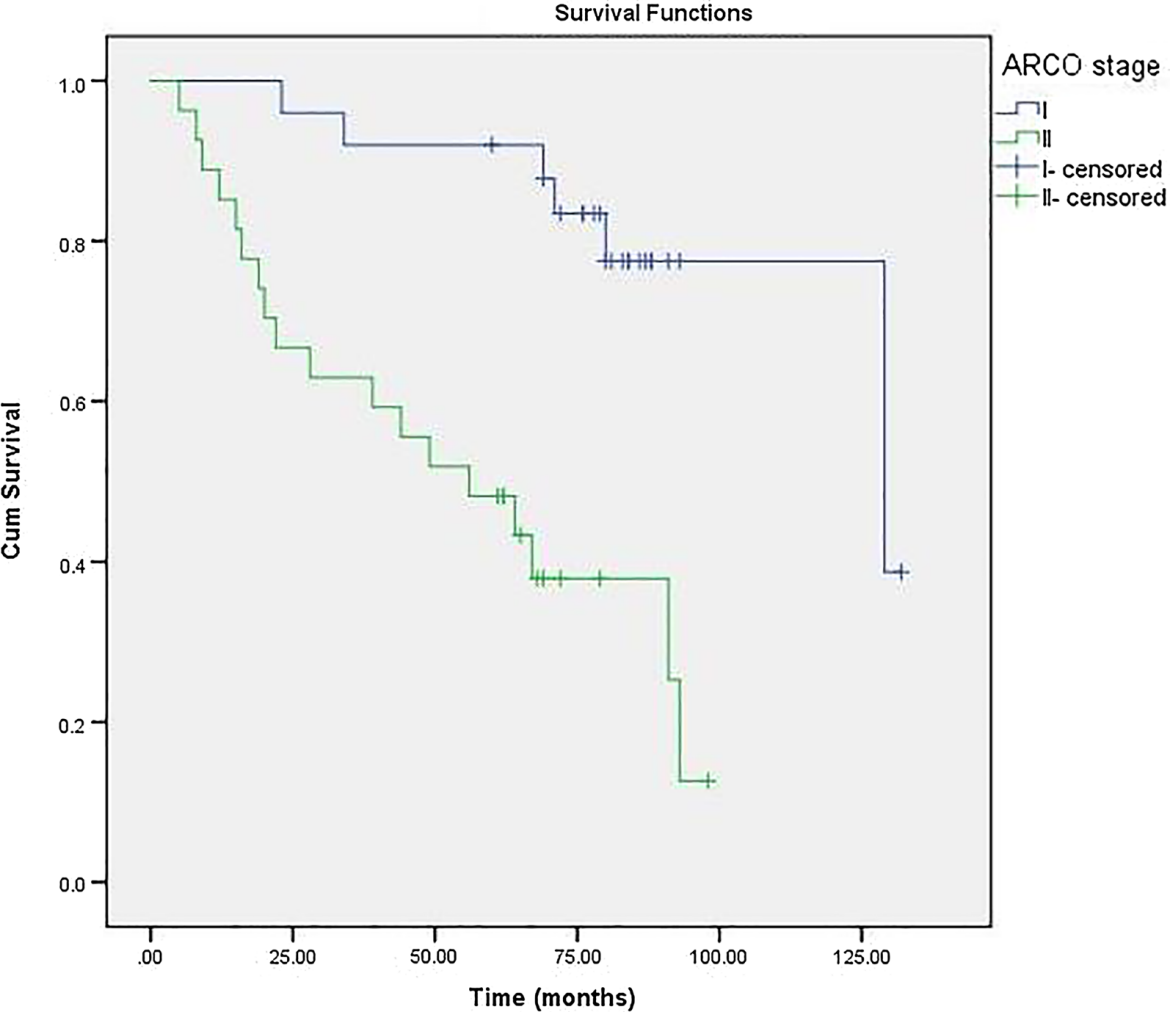
The survival time between Association Research Circulation Osseous (ARCO) stages I and II. The survival time was significantly shorter in the latter (*P* < 0.001)

**Fig. 6:**
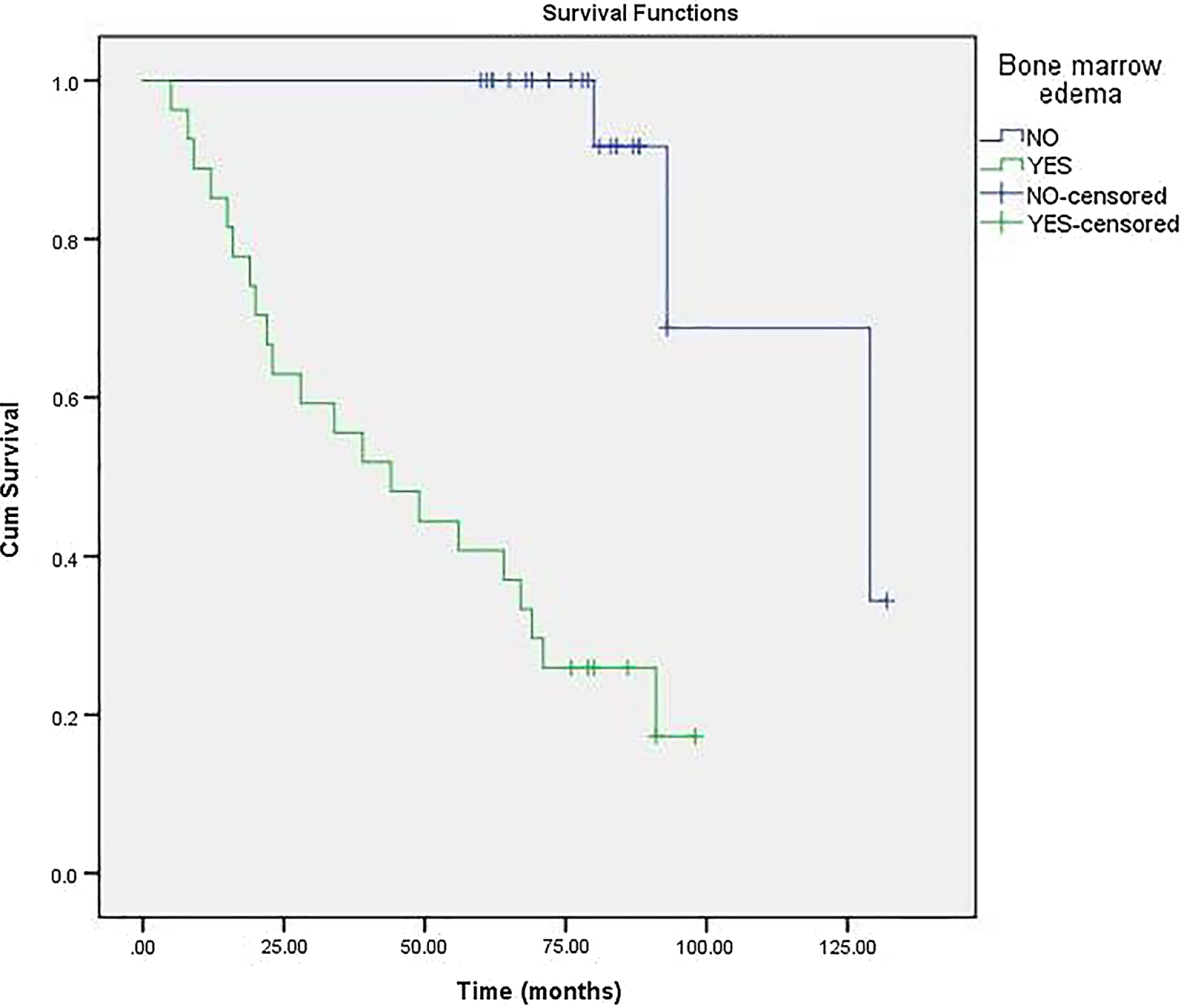
The survival time between bone marrow edema groups (YES or No). The survival time was significantly shorter in patients with bone marrow edema (*P* < 0.001)

For 52 hips with porous tantalum rod implantation, THA was necessary in 24 hips (19 patients). This implies a survival rate of 52.9% after porous tantalum rod implantation of osteonecrosis intervention. The 6-year cumulative survivorship of the porous tantalum rod implantation of osteonecrosis intervention was 60.0% (95% confidence interval [CI], 46.28%–73.72%) (Fig. 3). On the basis of this Cox proportional-hazards analysis, Kaplan–Meier survival curve was further drawn by stratification according to age (Fig. 4), ARCO stage (Fig. 5) and bone marrow edema (Fig. 6). The mean time of THA was (44.3 ± 32.8) months. Patients who need THA: 13 males, 6 females; 14 unilateral, 5 bilateral; 11 Corticosteroids hips, 11 alcoholic hips, 2 idiopathic hips; 5 in ARCO I stage and 19 in ARCO II stage. Before operation, 21 hips with Harris hip score < 80, 3 hips with Harris hip score ≥ 80; 5 hips with age ≤ 40, 19 hips with age > 40; 19 hips with bone marrow edema. The average age was (46.1 ± 6.4) years old, the average body mass index was (25.3 ± 4.9) kg/m^2^, the average operation time was (55.9 ± 8.7) min, the average amount of bleeding was (97.0 ± 28.0) ml, the average Harris hip score before porous tantalum rod implantation was (70.3 ± 6.2), and the average Harris hip score at the last time before THA was (57.1 ± 7.6).

The proportion of patients with different ARCO stage classification to THA was different. There was a significant difference between the two groups (x^2^ = 8.421, *P* < 0.05). The proportion of patients over 40 years old who needed THA was higher than those under 40 years old (x^2^ = 7.590, *P* < 0.05). The proportion of hips with bone marrow edema to THA was higher than that without bone marrow edema (x^2^ = 8.421, *P* < 0.05). 44.2% (13/30) of the male patients needed THA, and 55.6% (7/12) of the female patients needed THA. There was no significant difference between the sexes (x^2^ = 0.773, *P* > 0.05). There was no significant difference in the conversion to THA due to different etiology (hormone, alcohol, idiopathic) (x^2^ = 1.794, *P* > 0.05), unilateral or bilateral (x^2^ = 0.120, *P* > 0.05), preoperative Harris hip score ≥ 80 (x^2^ = 0.285, *P* > 0.05). (Table 3).

**Table 3:** Comparison of different groups Conversion to THA (%)

Classification		Conversion to THA	X^2^	P value
ARCO stage	I	5/22	8.421	0.004*
	II	19/30		
Bone marrow edema	Yes	19/30	8.421	0.004*
	No	5/22		
Age	> 40	20/33	7.590	0.006*
	≤ 40	4/19		
Etiology	Corticosteroids	11/21	1.794	0.408
	Alcoholism	11/23		
	Idiopathic	2/8		

*Denotes significant difference between groups (P < 0.05)

Cox proportional-hazards analysis revealed that ARCO stage (P = 0.017), Bone marrow edema (*P* = 0.006) and Age > 40 years (*P* = 0.043) were independent risk factors for conversion to THA; While conversion to THA were not correlated with Bilateral disease, Corticosteroids intake, Harris hip score ≥ 80 points, gender and osteonecrotic lesion ≥ 30%. (Table 4).

**Table 4:** The results of Cox proportional-hazards model for conversion to THA

Variable selected	B	SE	Wald	P value	95% CI
ARCO stage	1.991	0.835	5.689	0.017*	1.426–37.567
Bilateral disease	0.352	0.504	0.487	0.485	0.529–3.817
Bone marrow edema	3.531	1.279	7.627	0.006*	2.787–418.683
Osteonecrotic lesion ≥ 30%	1.873	1.100	2.902	0.088	0.754–56.173
Age > 40 years	3.023	1.494	4.096	0.043*	1.100–383.818
Harris hip score ≥ 80 points	− 13.182	404.806	0.001	0.974	0.000~
Gender	0.292	1.373	0.045	0.832	0.091–19.743
Corticosteroids intake	0.334	1.708	0.038	0.845	0.049–39.743

*Indicate the significant P value. *B* regression coefficient, *SE* standard error

### Postoperative complications

One patient developed deep infection after porous tantalum rod implantation at 2 months postoperatively. The result of bacterial culture was Staphylococcus epidermidis infection. The patients were treated with one-stage tantalum rod removal and antibiotic cement chain removal, and sensitive antibiotics were used after the operation, followed by two-stage THA. No postoperative complications such as femoral neck fracture and intertrochanteric fracture were found in other patients.

## Discussions

Porous tantalum rod implantation, as a kind of hip joint preserving operation, has good biocompatibility, elastic modulus similar to fibula, subchondral bone support and core decompression effect, and has achieved good short-term effect in clinic [17]. However, the long-stage effect of porous tantalum rod implantation is uncertain. Porous tantalum rod implantation in the treatment of femoral head necrosis failed and convert to THA has been successively reported [18, 19].

The prognosis of ONFH is related to age, bone marrow edema, corticosteroids intake and preoperative stage [10, 20]. The results of this study show that the prognosis of porous tantalum rod implantation is related to the age of patients, bone marrow edema and ARCO stage before operation. The operative effect of hip joint preservation in osteonecrosis of femoral head is related to clinical stage. When the femoral head collapses before operation, the prognosis is poor. Veillette et al. [8] followed up 58 patients with porous tantalum rod implantation and found that in 49 Steinberg stage II, 6 (12%) needed THA, and in 8 Steinberg stage III, 3 (38%) needed THA. Patients in stage III were more likely to fail than those in stage II. Zhao et al. [21] also pointed out that the survival rate of patients with ARCO IV stage (63.6%) was significantly lower than that of patients with ARCO II stage (95%) and ARCO III stage (92%) (*P* < 0.05). The results of this study showed that the survival rate of patients with ARCO stage II was lower than that of patients with stage I, and the failure rate was higher, and the ARCO stage was a risk factor for conversion to THA. Florkemeier et al. [22] followed up 19 patients (23 hips) who underwent porous tantalum rod implantation combined with core decompression for an average of 1.45 years. Although the patients included in the study were patients with early necrosis of the femoral head in ARCO I stage and II stage, the overall survival rate was only 44%. It was pointed out that tantalum rod combined with core decompression did not show obvious advantages compared with core decompression alone. The advantages of early weight-bearing in this operation may be of clinical significance. As a result, they did not recommend porous tantalum rod implantation for ONFH. A meta-analysis also concluded that the clinical effect of hip joint preservation surgery on patients with collapsed hip joint was poor [23].

ONFH is a progressive disease that easily affects young patients with age about 35 years [24].The young patients have a good prognosis after hip conserving surgery. The follow-up results of Nadeau et al. [18] showed that the mean age at surgery of the patients who failed was (50.1 ± 12.1) years old, compared to a mean age of (36.8 ± 12.2) years old for the patients whose tantalum implant did not fail, with significant difference (*P* < 0.05). Age is one of the prognostic factors of porous tantalum rod implantation. Tsao et al. [7] also pointed out that the older patients were prone to failure after porous tantalum rod implantation. Liu et al. [19] followed up 44 patients (57 hips) with modified porous tantalum rod implantation for an average of 44.8 months, and failed 11 hips (19.3%). The preoperative age did not affect the survival rate of the patients, and had nothing to do with the prognosis of the operation. Previous literature found that patients older than 35/50 years of age were more likely to receive THA during the postoperative follow-up period [10, 20]. The follow-up results of this study showed that patients with age > 40 years old had higher failure rate and lower survival rate, and age was a risk factor for THA, suggesting that patients' age was one of the factors affecting the effect of porous tantalum rod implantation.

Previous studies have shown that bone marrow edema is a poor prognostic signal because it occurs after the onset or deterioration of hip pain and is associated with subsequent collapse of the femoral head, which may indicate progression to ONFH [14, 15, 25]. Iida et al. [26] reported that bone marrow edema did not exist in the initial MR imaging of osteonecrosis, and concluded that bone marrow edema should be considered as a sign of possible progression to advanced osteonecrosis. According to Ito et al. [16], the final imaging stage of 28 hip patients with bone marrow edema was significantly more advanced than that without bone marrow edema. Bone marrow edema is closely related to necrosis volume and is the most important risk factor for the aggravation of hip joint pain. Consistent with these studies, this study determined that bone marrow edema is the most important independent prognostic factor associated with THA demand. Although the number of bone marrow edema in the hip is limited in the present study, our results show that the survival rate of the hip with bone marrow edema is significantly lower than that without bone marrow edema.

Our study retrospectively analyzed the medium and long-stage survival data of patients with ONFH treated with porous tantalum implant, and evaluated its clinical and imaging results. In addition, we analyzed the effects of porous tantalum implant on ONFH and related factors that led to the conversion of porous tantalum implant to THA, and evaluated the medium and long-stage efficacy of porous tantalum implant in the treatment of early ONFH. We found that the medium and long-stage clinical effect of porous tantalum rod implantation in the treatment of ONFH is not as satisfactory as we expected, and the osteogenic activity of tantalum rod in the femoral head is limited. ARCO stage, age and bone marrow edema were risk factors for the failure of porous tantalum rod implantation to THA. This study has important guiding significance for the treatment of patients who underwent porous tantalum implant in the past and later underwent THA.

We acknowledge some limitations of this study. First, this is a nonrandomized retrospective study of patients treated by different surgeons in different institutions and our statistical findings are also limited in power because of the small number of subjects. In the Cox proportional hazard model, occur in only 24 cases, theoretically only two can be used as explanatory variables. A larger investigation with more patients experiencing subsidence of the stem would be necessary to provide greater statistically significant information on the reason related to the failure risk for this finding. Second, the study failed to establish a control group to evaluate whether porous tantalum implants are superior to other head saving surgery. And the follow-ups, and thus postoperative score recording, were not done at the same time interval with respect to the surgery, making the results not as reproducible and precise as they could have been.

## Conclusion

The medium and long-stage clinical effect of porous tantalum rod implantation in the treatment of ONFH is not as satisfactory as we expected. ARCO stage, age and bone marrow edema were risk factors for the failure of porous tantalum rod implantation to THA.

## Data Availability

Because the study's data sets are included in ongoing research, Part of data sets used and analyzed in the study are available on reasonable request from corresponding authors.

## Abbreviations

THA: Total hip arthroplasty
ONFH: Osteonecrosis of the femoral head
ARCO: Association Research Circulation Osseous
